# Assessing the *Scholar* CanMEDS role in residents using critical appraisal techniques

**Published:** 2013-03-31

**Authors:** Aliya Kassam, Tyrone Donnon, Michèle Cowan, Joanne Todesco

**Affiliations:** University of Calgary

## Abstract

**Background:**

In this brief report, we describe two ways in which we assessed the *Scholar* CanMEDS role using a method to measure residents’ ability to complete a critical appraisal. These were incorporated into a modified OSCE format where two stations consisted of 1) critically appraising an article and 2) critiquing an abstract.

**Method:**

Residents were invited to participate in the CanMEDS In-Training Exam (CITE) through the Office of Postgraduate Medical Education. Mean scores for the two *Scholar* stations were calculated using the number of correct responses out of 10. The global score represented the examiner’s overall impression of the resident’s knowledge and effort. Correlations between scores are also presented between the two *Scholar* stations and a paired sample *t*-test comparing the global mean scores of the two stations was also performed.

**Results:**

Sixty-three of the 64 residents registered to complete the CanMEDS In-Training Exam including the two *Scholar* stations. There were no significant differences between the global scores of the *Scholar* stations showing that the overall knowledge and effort of the residents was similar across both stations (3.8 vs. 3.5, *p* = 0.13). The correlation between the total mean scores of both stations (inter-station reliability) was also non-significant (*r* = 0.05, *p* = 0.67). No significant differences between senior residents and junior residents were detected or between internal medicine residents and non-internal medicine residents.

**Conclusion:**

Further testing of these stations is needed and other novel ways of assessing the *Scholar* role competencies should also be investigated.

## Introduction

Postgraduate educators are held responsible to use the Canadian Medical Education Directives for Specialists (CanMEDS) roles framework from the Royal College of Physicians and Surgeons of Canada as the basis for developing medical curricula and measurement tools to assess residents throughout their training programs. The seven CanMEDS roles are *Medical Expert* (the central role), *Communicator, Collaborator, Health Advocate, Manager, Scholar* and *Professional*. 1 At present the RCPSC encourages resident training programs to teach and evaluate the CanMEDs roles, however, there are no current standards or practices in place as to the best method for developing such curricula and corresponding assessment. Furthermore, many resident training programs focus less on the “softer” roles such as Health Advocate, Scholar and Collaborator roles.^2^

### Residents as Scholars

As *Scholars*, resident physicians demonstrate a lifelong commitment to learning, creating and demonstrating as well as applying and translating medical knowledge. Ways of assessing the *Scholar* role in an evidence-based manner to ensure validity and reliability are scarce. Scholarly activity has been assessed in an Objective Structured Clinical Exam (OSCE) stations that required medical students to ask a clinical question, perform a literature search and evaluate the results of the search.^3^–^4^ Jefferies et al.^5^ assessed the *Scholar* role in residents in an OSCE,; however, only the teaching competency of the *Scholar* role was assessed. Results showed that second-year candidates scored higher than first-year candidates and interstation reliability (Cronbach’s alpha) for the two Scholar stations was very low at α = 0.08. Much of the existing research pertaining to residents as scholars centers on teaching^6^–^8^ while scholarly activity can represent a broad range of activity from teaching to journal clubs to resident’s own research.

Studies show that residents complete a variety of projects to fulfill the scholarly activity requirement; the evaluation of residents regarding these projects was not carried out so it is not known how much the residents actually learned from such activities or how they performed. For example, Rivera et al.^9^ looked at residents who completed a scholarly project during residency training. Seventy-three residents (53%) completed a questionnaire. Thirty-nine residents presented a clinical vignette, and 34 displayed a research abstract. It was found that residents participated in research for a variety of reasons, such as intellectual curiosity (73%), career development (60%), and to fulfill a mandatory scholarly activity requirement of their residency program (32%). The barriers were insufficient time (79%), inadequate research skills (45%), and lack of a formal research curriculum (44%). Sixty-nine percent of residents thought that research should be a residency requirement; however, residents were not assessed on any of these activities.

### Critical Appraisal

Critical appraisal skills are important for residents’ scholarly activity and should be assessed to ensure that residents are learning important skills and are able to apply them. Resident physicians need critical appraisal skills for their own research, participating in journal clubs, as well as for assessing clinical implications of treatments. There have been several studies of teaching critical appraisal skills and evidence-based medicine; however, these have shown mixed results as to whether they were successful in teaching critical appraisal skills. In a review, two studies that examined residents’ use of the literature were unable to demonstrate any positive changes after critical appraisal training.^10^ Other interventions have shown success in teaching evidence-based practice using journal clubs and teaching sessions.^11^,^12^

One aspect of critical appraisal which has not been addressed in the literature is resident’s ability to write and critique abstracts. Such a skill is important because residents will be expected to submit abstracts to conferences, and write them for peer-reviewed papers and funding applications. In this brief report, we describe two ways in which we assessed the *Scholar* role using a method to measure residents’ ability to complete a critical appraisal. These were incorporated into a modified OSCE format where two stations consisted of 1) critically appraising an article and 2) critiquing an abstract.

## Methods

### Participants

Sixty-three of the 64 residents registered to complete the CanMEDS In-Training Exam including the two Scholar stations. Residents were from different programs; however, the majority (63%) were from Internal Medicine. Sixty-eight percent (*n* = 42) of the residents were senior residents (4^th^ year and above).

The CanMEDS in-Training Exam (CITE) was held in March 2012. This exam consisted of 8 stations, 6 of which measured two alternating CanMEDS roles (e.g., Station #1 = primary role: *Professional* and secondary role *Health Advocate*). The remaining two stations focused on the *Scholar* role. The critical appraisal station included a paper from a high impact factor journal regarding a multi-center trial. The task of the resident was to find 5 strengths and 5 weaknesses of the study. The authors had set criteria for existing strengths and weaknesses in the trial; however, any strengths and weaknesses not identified *a priori* by the authors that were considered meritorious were given marks. Residents could obtain a maximum score of 10 with a maximum of 5 strengths and 5 weaknesses. If residents identified more strengths and weaknesses that were meritorious, once they reached a maximum score, no further marks were given.

For the other *Scholar* station, an existing structured abstract was modified and presented as an unstructured abstract with key findings excluded, lack of detail regarding the sample size and study method, improper use of statistical notation, and improper use of abbreviations. Residents were asked to find 10 ways that the abstract could be improved. If they provided more than 10 improvements they still only received the maximum score of ten. The authors had several improvements that were determined *a priori*. Again, if residents suggested an improvement that was judged to be meritorious but not already determined by the authors, they were given a mark for this. Residents were given ten minutes to complete these two stations. Resident performance was graded after all of the CITE stations were completed. Mean scores (SD) of each of the *Scholar* stations were computed along with global assessment scores of the resident’s overall performance. Mean scores represented the number of correct responses out of 10 for each station whereas the global score represented the examiner’s overall impression of the resident’s knowledge and effort. Global scores are based on a 5 point Likert scale (from 1 = Fails to meet expectations to 5 = Exceeds expectations). Correlations between scores are also presented between the two *Scholar* stations and a paired sample *t*-test comparing the global mean scores of the two stations was also performed.

## Results

There were no significant differences between the global scores of the Scholar stations showing that the overall knowledge and effort of the residents was similar across both stations (3.8 vs. 3.5, *p* = 0.13). The correlation between the total mean scores of both stations (inter-station reliability) was also non-significant (*r* = 0.05, *p* = 0.67). No significant differences between senior residents and junior residents were detected or between internal medicine residents and residents from other specialties. Table 1 shows the total mean and global mean scores of the Scholar stations.

## Discussion

Our results showed that the critical appraisal skills of residents, as part of the *Scholar* CanMEDS role, can be assessed using an OSCE formatted examination process. We found that residents obtained a higher score at the station which involved critically appraising an article when compared to critiquing an abstract which may reflect the efforts of resident training programs that have offered prior critical appraisal training through journal clubs or article review workshops. The lower score in the one station that involved the critique of an abstract points to a need for further training in this area given the need for residents to submit abstracts for conference presentations and funding applications during their residency program.

A limitation of this study however, is that we had only one examiner for each station and cannot provide any results in terms of inter-rater stringency or leniency. Both examiners, however, have an academic background and were involved in the design of the *Scholar* station. Future research should investigate the reliability of such stations having more than one examiner.

While we present two ways of assessing critical appraisal in residents, other novel ways of assessing the *Scholar* role competencies should be investigated since the breadth of scholarly activity spans beyond teaching to their ability to conduct research and dissemination of new knowledge.^9^ Resident training programs not only need to keep track of scholarly activities by residents, but incorporate how these activities are to be assessed in an evidence-based manner. Workshops covering a range of scholarly topics through PGME are important as they would provide an avenue for residents to learn and be assessed.

## Figures and Tables

**Table 1:** Total and global mean scores for the *Scholar* role stations

Scholar Station	Total Mean Score (SD)	Global Mean Score (SD)
Critical Appraisal of an Article	8.0 (1.5)	3.8 (0.8)
Abstract Critique	6.5 (2.7)	3.5 (1.4)
